# Genetic Comparison and Selection of Reproductive and Growth-Related Traits in Qinchuan Cattle and Two Belgian Cattle Breeds

**DOI:** 10.3390/ani15040608

**Published:** 2025-02-19

**Authors:** Xiaopeng Li, Peng Niu, Xueyan Wang, Fei Huang, Jieru Wang, Huimin Qu, Chunmei Han, Qinghua Gao

**Affiliations:** 1 College of Animal Science and Technology, Tarim University, Alar 843300, China; miexiaochi@163.com (X.L.);; 2 College of Life Science and Technology, Tarim University, Alar 843300, China; 3 Key Laboratory of Livestock and Grass Resources Utilization around Tarim, Ministry of Agriculture and Rural Areas (Co-Construction by Ministries and Provinces) & Construction Corps, Alar 843300, China

**Keywords:** linkage disequilibrium (LD), effective population size (Ne), fixation index (Fst), nucleotide diversity, enrichment analysis

## Abstract

This study investigates the genetic structure of Belgian Red (BR), Belgian Red and White (BWR), and Qinchuan (QinC) cattle, with a focus on identifying genes associated with reproductive functions, growth, and development. A total of 270 Belgian cattle (91 BR and 179 BWR) and 286 Qinchuan cattle were genotyped using the Illumina Bovine SNP 50K microarray. Data analysis was conducted using PLINK and Beagle 5.1 to estimate linkage disequilibrium (LD) and effective population size (Ne). Candidate SNP loci were identified by selecting the top 5% based on the weighted fixation index (Fst) and nucleotide diversity (θπ ratio), followed by gene annotation. The analysis revealed 160 candidate genes under selection between Qinchuan and Belgian Red cattle, and 98 candidate genes between Qinchuan and Belgian Red and White cattle. Key genes associated with reproductive functions, including *NFKBIA*, *PTHLH*, *UGT2B10*, *TRPC4*, and *ALOX5AP*, were identified. Additionally, genes involved in growth and muscle development were highlighted, particularly those influencing protein synthesis, fatty acid metabolism, and collagen synthesis. These findings provide valuable molecular markers for enhancing reproductive efficiency, growth, and meat production through genetic selection and selective breeding strategies.

## Simple Summary

This study explores the genetic factors that control reproduction in three cattle breeds, namely Belgian Red, Belgian White Red, and Qinchuan, by analyzing their population structures. Understanding these factors is important for improving cattle breeding programs, which can lead to healthier animals, better productivity, and more sustainable farming practices. The researchers identified specific genes that play a role in the reproductive processes of these cattle breeds. By examining the genetic makeup of these animals, the study provides insights into how certain genes influence fertility and overall herd performance. The findings could help farmers make informed decisions about breeding to improve livestock health and reproductive efficiency, ultimately contributing to the sustainability of cattle farming. This research can also support efforts to conserve genetic diversity in cattle populations, which is essential for ensuring long-term agricultural success.

## 1. Introduction

Belgian Red Bulls (BR) are an important regional breed in Belgium, but their low reproductive efficiency has placed them on the brink of extinction, which poses a significant challenge for sustainable cattle breeding [1]. Genetic bottlenecks and inbreeding within this population have further exacerbated these breeding difficulties [2,3]. On the other hand, Qinchuan cattle, a highly valued local breed in China, are recognized for their robust disease resistance, excellent adaptability, and stable breeding performance [4]. Given the endangered status of Belgian Red Bulls, a comparative study between these two breeds could offer valuable insights for enhancing reproductive performance in BR cattle. While breeds like Simmental and Holstein have been widely used in Chinese breeding programs, BR and its variant, Belgian Red and White (BWR), have not been systematically introduced or bred systematically in China. This study, therefore, focuses on comparing BR and BWR cattle with Qinchuan cattle to explore their genetic diversity and identify genes associated with reproductive traits. Through this comparison, we aim to identify variety-specific genetic variants that may potentially improve reproductive efficiency and growth and development, particularly through hybridization strategies.

Linkage disequilibrium (LD) enhances the accuracy of genomic association analyses and facilitates the identification of marker regions [5]. LD decay patterns offer critical insights into the evolutionary history of populations and are used to estimate the ancestral effective population size (Ne) [6]. Ne and other genetic events influence the extent of LD in a population, making LD a vital tool for understanding the selection pressures acting on specific breeds [7]. The Fst statistic is widely used to assess genetic variation between populations, typically calculated based on differences in gene frequencies through methods like AMOVA or SNP data analysis. Additionally, pi (nucleotide diversity) measures genetic variability within populations or species, representing the overall genetic diversity. Zhang et al. used data from the Illumina Ovine SNP50 chip to calculate LD r^2^ in 73 sheep from the Hetian region, inferring the effective population size of sheep. Their analysis revealed that LD was highly breed-specific in sheep, with Ne values comparable to those observed in other sheep breeds during specific periods of population decline [8]. Wang et al. utilized whole-genome sequencing data from Qinchuan cattle to examine selection signals [9]. Selection analyses based on Fst in Qinchuan, Red Angus, and Japanese Black cattle revealed genes associated with meat quality, such as *ARIH2*, *DACT1*, and *DNM2*.

This study aims to investigate the genetic architecture of Belgian Red Bulls and identify the key genes associated with reproductive traits, establishing a scientific basis for developing targeted breeding strategies.

## 2. Materials and Methods

### 2.1. Genotyping Data Sources

This study analyzed 286 Qinchuan cattle (NCBI GEO: PRJNA390539), 91 Belgian Red (BR), and 179 Belgian Red and White (BWR) cattle (Figshare: https://figshare.com/search?q=10.6084%2Fm9.figshare.17025086 accessed on 31 December 2024).

### 2.2. SNP Chip Quality Control and Filling

We extracted the GEO dataset Forward strand converted it to a format recognizable by the PLINK software, and then merged the files and performed quality control of the SNP data using the PLINK (version number 1.90) software. The following filters were applied to the data of samples and loci: (1) loci with SNP detection rate of less than 90% were excluded (-geno 0.1); (2) individual samples with a sample detection rate of less than 90% were excluded (-mind 0.1); and (3) minimum allele frequency greater than 5% (-maf 0.05). Beagle 5.1 https://faculty.washington.edu/browning/beagle (accessed on 10 February 2025) was used to populate the genome with fixed phases.

### 2.3. LD Calculation Method

Studying linkage disequilibrium (LD) decline provides insights into the history of population recombination. By analyzing LD between loci, it is possible to assess LD levels in the genomes of three beef cattle populations. In this study, we used PopLDdecay (v3.41) to calculate LD for SNP loci across the genome [10]. The LD value ranges from 0 to 1, with a higher r^2^ value indicating stronger linkage [11]. Perl scripts were employed to visualize the results and to plot the distribution of Mean_r^2^ across different groups.

### 2.4. Estimation of Effective Group Size

Effective population size (Ne) is a key parameter in population genetics, influencing the rates of genetic drift, inbreeding, and the efficiency of evolutionary forces such as mutation, selection, and migration. We estimated Ne using the SMC++ method [12], which models population size and split timing in PRS. This method incorporates a novel spline regularization scheme, which significantly reduces estimation errors.

To prepare the input files, we utilized the vcf2smc script from SMC++, converting each VCF file into the required SMC++ format. The analyses were conducted under initial conditions with a generation interval (-g) of 6 and a mutation rate of 1.26 × 10⁻^8^ per site per generation [13].

### 2.5. Diversity of Population Structure

Principal component analysis (PCA) based on variance normalized relationship matrix was performed on the data after quality control using Plink (V1.90) software, NJ matrix was calculated using VCF2Dis (https://github.com/BGI-shenzhen/VCF2Dis accessed on 10 February 2025) and evolutionary trees were generated using FastME (http://www.atgc-montpellier.fr/fastme/ accessed on 10 February 2025) to generate evolutionary trees.

### 2.6. Fixation Index

Fst is a statistical test measure of the degree of differentiation between populations, which is mainly used to study the degree of genetic variation between different populations as well as the population structure and population genetic diversity. It assesses the genetic differences between populations by calculating the mean square of error (MSG) for loci within a population and the mean square difference (MSP) for loci between populations. The formula for Fst is given below:

$$Fst=\frac{MSP-MSG}{MSP+(n_{c}-1)\;MSG}$$

where MSG denotes the mean square of the error of the detected intra-population loci, MSP denotes the mean square of the detected inter-population loci, and N is the corrected inter-population mean sample size. In this study, the VCFtools software (version 1.90) was used to calculate the SNP loci left by the Fst statistic calculation, which is an estimate based on Weir and Cockerham’s [14].

### 2.7. Nucleotide Diversity (θπ Ratio) Analysis

Nucleotide diversity (PI) is the value obtained by randomly selecting a segment of DNA sequences from multiple samples in a given population and then averaging the bases of these sequences at the same site. It is an important measure of nucleotide polymorphism within a population. The formula for calculating nucleotide diversity is given below:

$$PI=\sum_{j=i}^{s}h_{j}$$

where *S* denotes the number of segregating sites and h_j_ denotes the heterozygosity of the *j*th segregating site. The PI values can be obtained by calculating the nucleotide diversity of the populations using the VCFtools software. In this study, the degree of selection of this SNP locus from a genomic perspective was calculated by counting the ratio of PI on the same SNP locus in Qinchuan cattle and Belgian Red and Belgian Red and White cattle. When the ratio is smaller, it indicates that the nucleotide diversity of the Qinchuan cattle population is higher compared with that of Belgian cattle, which indicates that the locus is subjected to a smaller and larger degree of selection on the genome of Qinchuan cattle.

### 2.8. Enrichment Analysis of Candidate Genes

The top 5% of the screened loci were annotated as selected loci with reference to bovine genome UMD_3.1.1. Referring to the NCBI database (http://www.ncbi.nlm.nih.gov/gene accessed on 10 February 2025), the R language clusterProfiler package was used for the autosomal enrichment of candidate genes for GO enrichment analysis: Biological Process, Cellular Component, Molecular Function, and KEGG pathway enrichment analysis [15].

## 3. Results

### 3.1. Population Structure

**Figure 1:**
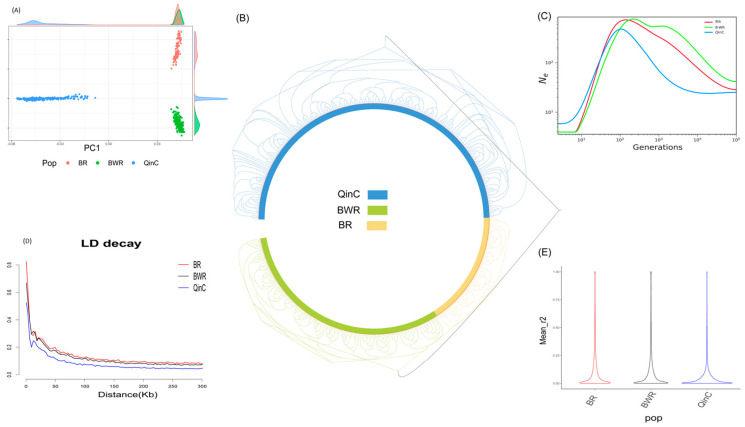
(**A**) The PCA results of three cattle populations with PC1 on the x axis and PC2 on the y axis; (**B**) Evolutionary tree of the three bovine populations, blue for QinC, green for BWR, and yellow for BR; (**C**) genome-wide LD (r^2^) to estimate Ne in different populations; (**D**) LD decay plots of three cattle populations where the X axis represents the distance, the Y axis represents the chain imbalance coefficient, red represents BR, black represents BWR, blue represents QinC; (**E**) LD(r2) distribution pattern of three different populations.

After the quality control of the SNP chip, 35078 high-quality SNP loci were retained, covering the genotypic data of 556 cattle. PCA was performed on the three cattle populations, and the results showed clear differentiation between the Chinese native Qinchuan (QinC) and Belgian Red (BR) and Belgian Red and White (BWR) cattle on PC1. Additionally, the three populations were also distinctly separated on PC2, indicating genetic differences between the selected breeds (Figure 1A). The NJ tree further confirmed these differences, with individuals from the three breeds clustering into separate branches (Figure 1B). Linkage disequilibrium (LD) analysis revealed that LD decay was the fastest in Qinchuan cattle, indicating higher genetic diversity and a lower degree of domestication in this population (Figure 1D). Moreover, the r^2^ statistics for LD showed that the QinC population had more SNP loci with an r^2^ value equal to 0 compared to the BR and BWR populations, further supporting this finding (Figure 1E). The effective population size (Ne) estimation indicated that the population size reached its peak between 100 and 250 generations ago, with a significant decline in Ne values in the last 10 generations, reflecting fluctuations in population size across different periods (Figure 1C).

### 3.2. Selective Sweeps

#### 3.2.1. Fixation Index Results

**Figure 2:**
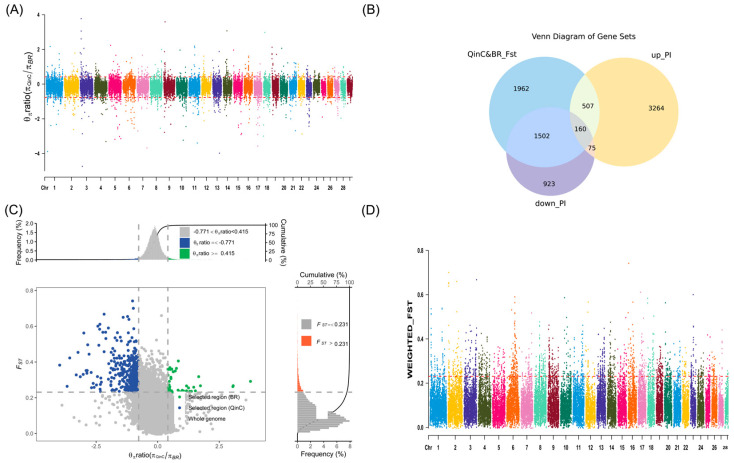
A multi-panel visualization of selection signals in the QinC and BR populations based on different genetic selection metrics. The figure consists of four subplots (**A**–**D**), each illustrating different aspects of selection patterns: (**A**) Chromosomal distribution of selection degree (θπ ratio). X axis: Represents different chromosomes, showing genome-wide selection patterns across the chromosomes. Y axis: Represents the selection degree values θπ ratio (πQinC /πBR), which compares nucleotide diversity (π) between QinC and BR populations. Red-dashed lines: The upper-dashed line (0.415) and lower-dashed line (−0.771) indicate the top and bottom 2.5% threshold values for θπ ratio, which define regions under selection. Data points above the upper threshold represent genomic regions that experienced strong positive selection in QinC (higher diversity in QinC relative to BR). Data points below the lower threshold indicate genomic regions under stronger selection in BR (lower diversity in QinC compared to BR). (**B**) Venn diagram of selected genes. Three main categories are depicted: Genes identified in the top 5% of Fst-selected regions (representing highly differentiated genes between QinC and BR); Genes within the top 2.5% of θπ ratio upregulated genes (genes that show higher nucleotide diversity in QinC compared to BR, suggesting positive selection in QinC). Genes within the top 2.5% of θπ ratio downregulated genes (genes that show lower nucleotide diversity in QinC compared to BR, suggesting positive selection in BR)**.** Overlap among these categories: The intersection of these sets highlights genes that are subject to both differentiation and strong selection, making them potential candidates for adaptive evolution. (**C**) Scatterplot of θπ ratio vs. Fst values. X axis: Represents θπ ratio (πQinC /πBR), showing the relative selection strength between the two populations. Y axis: Represents Fst values, measuring genetic differentiation between QinC and BR populations at each genomic locus. Dot colors indicate population-specific selection: Blue dots: Represent regions predominantly under selection in QinC. Green dots: Represent regions predominantly under selection in BR. Higher Fst values suggest stronger genetic divergence between populations, while extreme θπ ratio values indicate selection pressure acting more intensely on one population. (**D**) Chromosomal distribution of WEIGHTED_Fst Values. X axis: Represents different chromosomes, similar to panel (**A**). Y axis: Represents WEIGHTED_Fst values, a metric indicating genetic differentiation across different genomic regions. Red-dashed line (threshold = 0.231): Represents the top 5% threshold of WEIGHTED_Fst values. Genomic regions above this threshold are significantly differentiated between QinC and BR populations, indicating strong selective pressure leading to population-specific divergence.

**Figure 3:**
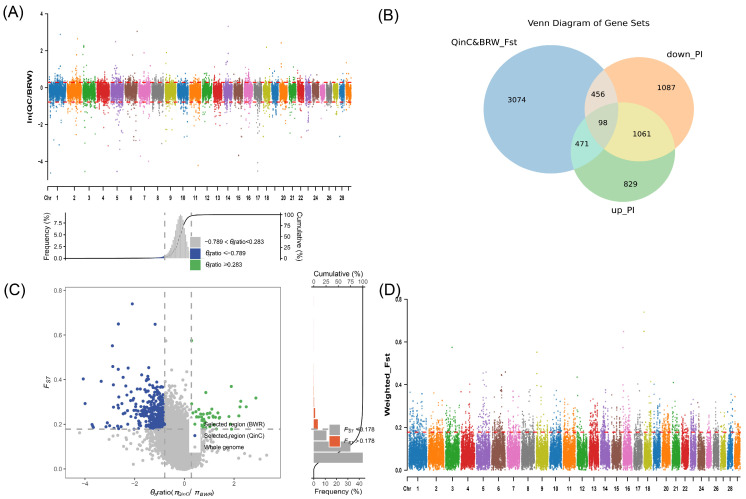
A multi-panel visualization of selection signals in the QinC and BWR populations based on different genetic selection metrics. The figure consists of four subplots (**A**–**D**), each illustrating different aspects of selection patterns. (**A**) Chromosomal distribution of selection degree (θπratio) **X axis:** Represents different chromosomes, showing genome-wide selection patterns. Y axis: Represents the selection degree θπratio (πQinC/πBWR), which compares nucleotide diversity (π) between QinC and BWR populations. Red-dashed lines: The upper threshold (0.283) and lower threshold (−0.789) mark the top and bottom 2.5% quantiles of θπ ratio, defining regions under selection. Data points above the upper threshold indicate genomic regions under strong positive selection in QinC (higher diversity in QinC relative to BWR). Data points below the lower threshold indicate genomic regions under stronger selection in BWR (lower diversity in QinC relative to BWR). (**B**) Venn diagram of selected genes. This panel depicts the overlap among three categories of selected genes: Genes in the top 5% of Fst-selected regions, representing loci with high genetic differentiation between QinC and BWR (blue). Genes in the top 2.5% of θπ ratio upregulated genes, showing higher nucleotide diversity in QinC compared to BWR, suggesting positive selection in QinC (green).Genes in the top 2.5% of θπ ratio downregulated genes, showing lower nucleotide diversity in QinC compared to BWR, suggesting positive selection in BWR (orange). Overlap among these categories: The intersection of these sets highlights genes subject to both strong differentiation (Fst) and selection (θπratio). These genes are potential candidates for adaptive evolution in either population. (**C**) Scatterplot of θπratio vs. Fst values. This panel visualizes the relationship between genetic differentiation (Fst) and nucleotide diversity ratio (θπratio) in the QinC and BWR populations, helping to identify regions under selection. X axis: Represents the θπ ratio (πQinC/πBWR), reflecting the relative selection strength between the two populations. Y axis: Represents Fst values, quantifying genetic differentiation between the populations. Higher Fst values indicate strong genetic divergence, suggesting that selection may have driven population-specific allele frequency shifts. Dot colors indicate selection patterns in different populations: Blue dots: Represent regions predominantly under selection in QinC. Green dots: Represent regions predominantly under selection in BWR. Higher Fst values indicate stronger genetic divergence between populations, while extreme θπ ratio values suggest intense selection pressure acting on one population. (**D**) Chromosomal distribution of WEIGHTED_Fst Values. X axis: Represents different chromosomes, as in panel (**A**). Y axis: Represents WEIGHTED_Fst values, measuring genetic differentiation across genomic regions. Red-dashed line (threshold = 0.178): Marks the top 5% quantile of WEIGHTED_Fst values. Genomic regions above this threshold show significant differentiation between QinC and BWR, indicating strong selective pressure driving population divergence.

We set the sliding window size to 500 kb and the step size to 50 kb. We arranged the Fst values of QinC and BR, BWR in descending order, and selected the highest 5% as the selection region, respectively. After the Fst screening, 4131 versus 4355 genes were identified, respectively (Figure 2D and Figure 3D, Supplementary Table S2).

#### 3.2.2. Nucleotide Diversity (θπ Ratio) Analysis

Nucleotide diversity *p* values of SNP loci of the QinC population were divided with the *p* values of the BR population, and were sorted in ascending order by taking e as the base number, selecting two different populations subject to the selection of loci, and selecting the high and low values of 2.5% each as the selection regions, and 4006 and 2660 genes were found, respectively (Figure 2B, Supplementary Table S3). The nucleotide diversity *p*-values of SNP loci in the QinC population were divided by the *p*-values of the BWR population, the logarithms were sorted in ascending order, the 2.5% higher and lower values were selected as the selection regions, and 2459 versus 2783 genes were found, respectively (Figure 3B, Supplementary Table S4).

#### 3.2.3. Enrichment Analysis of Candidate Genes Results

For the QinC and BR populations, 160 intersecting candidate genes were obtained after the intersection analysis of the genes screened by Fst and PI (Figure 2B,C, Supplementary Material Table S5), and for enrichment analysis, we found pathways related to growth and development as well as pathways related to reproduction (Table 1).

**Figure 4:**
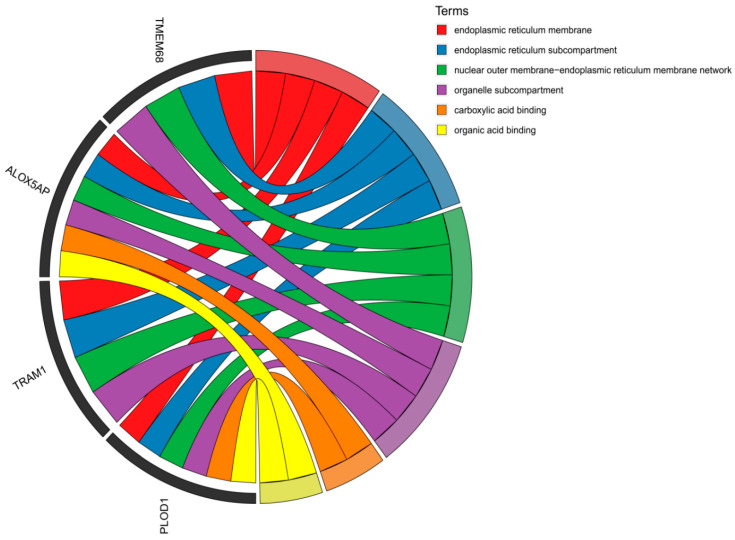
A circular chord diagram illustrating the functional enrichment of the top 5% candidate genes in the BWR population. This visualization highlights the relationships between key genes (left side) and their corresponding enriched pathways (right side).

For the QinC and BWR populations, after the intersection analysis of the genes screened by Fst and PI, we obtained 98 intersection candidate genes, (Figure 3B,C, Supplementary Material Table S5), and for the enrichment analysis, we found some pathways related to growth and developmental pathways (Figure 4), for example: (GO:0031406~carboxylic acid binding, GO:0043177~organic acid binding).

## 4. Discussion

### 4.1. Analysis of Population Structure

Principal component analysis (PCA) was performed on 556 cattle to cluster the three populations clearly separated (Figure 1A), using PC1, PC2 it can be seen that the three groups possess a clear differentiation. Qinchuan cattle were clearly separated compared to the other groups, while Belgian Red and Belgian Red and White cattle were clustered into one group on PC1 which is consistent with the genetic recognition, the PCA results were consistent with the results of the evolutionary tree, the two groups (BR, BWR) of Qinchuan cattle and Belgian cattle possessed completely different evolutionary branches, starting from the root node and later on in different directions (Figure 1B).

Ne values were estimated for the QinC, BR, and BWR populations based on a mutation rate of 1.26 × 10⁻^8^, with a generation interval of 6 years. The analysis revealed an increase in Ne around generation 104, which corresponds to approximately 624 years ago (assuming 6 years per generation). This period aligns with the post-domestication population expansion, likely driven by advancements in agricultural practices rather than the initial domestication event, which occurred thousands of years earlier [16]. In the past 100 generations, Ne values began to decline, potentially due to industrialization and shifts in agricultural production. The highest Ne values were observed between 100 and 250 generations ago, likely reflecting an increase in population size due to agricultural demands (BWR: 767.43, BR: 738.71, QinC: 481.51). In the 10 most recent generations, Ne values stabilized at minimal levels (BWR: ~4, BR: ~4, QinC: ~6), indicating a genetic bottleneck in the Belgian breeds compared to QinC. These results are consistent with the long-term isolation of these breeds, which have been separated for thousands of years (Figure 1C, Supporting Material Table S1).

The distance between LD markers was set between 0 and 300 kb. The average r^2^ decreased with increasing physical distance (Figure 1D). From the decay rate, it can be seen that BR decayed the fastest when the distance between markers was 0–10 kb, proving that the genetic diversity of the population was high, while the BWR was close to BR, proving the fact that these two populations were genetically closer together, and compared to the BR and BWR populations, QinC decayed slower, proving a high degree of domestication.

### 4.2. Reproductive Performance-Related Genes

**Figure 5:**
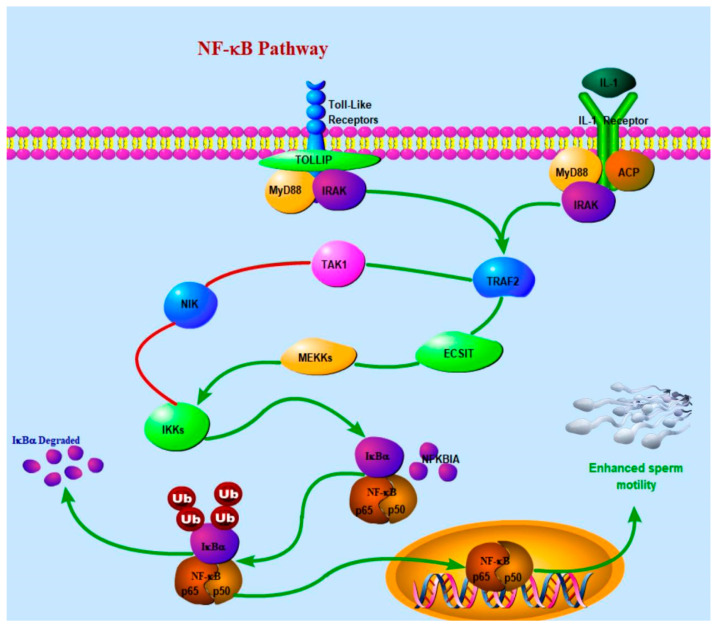
The red line in the figure represents the IKBA phosphorylation process, and the inhibition of the phosphorylation process improves sperm motility and thus enhances the reproductive performance of bulls.

Reproductive efficiency in large beef cattle is a critical concern in agricultural production [17]. Belgian cattle are highly valued by farmers and ranchers for their superior meat yield and body condition [18]. However, the endangered status of Belgian Red Cattle has raised significant concerns. Lucas Marian et al. reported that Belgian Red Cattle are susceptible to Neisseria canis infection, which may contribute to reproductive disorders and increased abortion rates within the herd [19]. To better understand the genetic basis of reproductive performance, we investigated key signaling pathways associated with reproduction in Belgian cattle. Our analysis identified NFKBIA as a crucial gene involved in the KEGG pathway bta04926 (relaxin signaling pathway) (Table 1). NFKBIA encodes a member of the NF-κB inhibitor family, which contains multiple ankyrin repeat domains. Notably, the inhibition of IκBα phosphorylation has been shown to significantly enhance sperm motility [20]. Moreover, mutations in NFKBIA are associated with ectodermal dysplasia [21], suggesting that its expression may influence reproductive performance (Figure 5). Beyond *NFKBIA*, several other genes identified in our analysis play key roles in both reproduction and growth. *PTHLH* encodes a neuroendocrine peptide that regulates cellular and organ growth, development, migration, differentiation, and survival [22]. It also plays a critical role in mammary gland and bone development, contributing to its influence on reproductive function in cows. In beef cattle, *PTHLH* regulates endochondral osteogenesis and epithelial–mesenchymal interactions, which are essential for bone formation and homeostasis [23]. *UGT2B10* (Uridine 5′-diphospho-glucuronosyltransferase 2B10) is a UDP-glucuronosyltransferase primarily involved in the N-glycosylation of drugs and xenobiotics. This gene regulates estrogen metabolism and clearance during the reproductive cycle, thereby influencing reproductive hormone levels and fertility in female animals [24,25]. The *TRPC4* gene encodes a protein expressed in gonadotropin-releasing hormone (GnRH) neurons [26,27]. This protein regulates the excitability of GnRH neurons, which control the secretion of gonadotropic hormones from the pituitary gland [28]. Through the formation of calcium-permeable cation channels [29,30], *TRPC4* influences the membrane potential of GnRH neurons, thereby modulating their activity [31]. Alterations in *TRPC4* expression or mutations may disrupt normal GnRH neuron function, ultimately affecting reproductive hormone release and the overall reproductive system function. *ALOX5AP* encodes a 5-lipoxygenase-activating protein, which plays a key role in the prostaglandin synthesis pathway. This protein is involved in arachidonic acid metabolism, catalyzing the conversion of arachidonic acid (AA) to leukotriene A4 (LTA4), a critical step in prostaglandin biosynthesis. The overexpression of *ALOX5AP* has been associated with inflammatory and immune response processes [32], which may have indirect implications for reproductive health. Collectively, these findings highlight the involvement of multiple genes in reproductive regulation, many of which also influence growth and development. Further research is necessary to elucidate the precise molecular mechanisms underlying their roles in reproductive performance.

### 4.3. Analysis of Genes Related to Growth, Development, and Meat Production

In our study, enrichment analysis highlighted several endoplasmic reticulum (ER)-related pathways crucial for growth, development, and meat production [33]. Genes such as TMEM68, ALOX5AP, TRAM1, and PLOD1 are involved in these pathways, playing essential roles in protein synthesis, folding, and trafficking, which are vital for muscle growth and meat formation [34]. Furthermore, ALOX5AP and PLOD1 also participate in pathways related to fatty acid metabolism and collagen synthesis, which are key for muscle development and meat quality [35,36]. These findings suggest that ER-related genes are important for muscle growth and meat quality. By comparing Belgian Red (BR) and Belgian Red and White (BWR) cattle with Qinchuan cattle, we identified genetic traits that could improve Qinchuan’s growth and meat characteristics through crossbreeding. Despite environmental differences between China and Belgium, this genetic comparison offers a strategy to introduce beneficial traits into breeding programs. Future studies using whole-genome sequencing (WGS) will help validate these findings, and including more cattle breeds from diverse regions will provide deeper insights into breed-specific adaptations, aiding in the preservation of genetic diversity.

## 5. Conclusions

This study highlights the importance of genetic diversity and reproductive efficiency in the conservation of endangered breeds, particularly Belgian Red cattle. Our results suggest that comparing the reproductive performance and genetic differences between Qinchuan cattle and Belgian Red cattle can significantly enhance both reproductive outcomes and genetic robustness. The identification of key reproductive genes provides new insights into improving cattle fertility through genetic selection and crossbreeding strategies. 

Moreover, while environmental differences between China and Belgium exist, the genetic analysis allows for a comparative approach to identify beneficial traits that could potentially be introduced into breeding programs. Future research should focus on expanding genomic studies with whole-genome sequencing (WGS) data to validate the findings, as SNP beadchip data have moderate genome coverage. Additionally, incorporating a wider range of cattle breeds from different geographic regions would allow for a more comprehensive understanding of breed-specific adaptations. These insights could shape future conservation strategies and contribute to the preservation of livestock genetic diversity. 

## Figures and Tables

**Table 1:** QinC and BR intersection candidate genes enriched for pathways associated with growth, development, and reproduction.

Function	Term	Pvalue	Gene
Growth and development	GO:0061035~regulation of cartilage development	0.021454098	*PTHLH*
	GO:0002062~chondrocyte differentiation	0.044240533	*PTHLH*
	GO:0030282~bone mineralization	0.056316761	*PTHLH*
	GO:0061448~connective tissue development	0.091748173	*PTHLH*
	bta00040~Pentose and glucuronate interconversions	9.53677E-05	*UGT2B10*
Reproductive correlation	GO:0045596~negative regulation of cell differentiation	0.163777049	*PTHLH*
	bta04929~GnRH secretion	0.159818885	*TRPC4*
	bta04917~Prolactin signaling pathway	0.203839237	*GCK*
	bta04926~Relaxin signaling pathway	0.298730333	*NFKBIA*

## Data Availability

The original contributions presented in this study are included in the article/Supplementary Material. Further inquiries can be directed to the corresponding author(s).

## Supplementary Materials

The following supporting information can be downloaded at: https://www.mdpi.com/article/10.3390/ani15040608/s1, Supplementary Material Table S1. SMC++ Ne; Supplementary Material Table S2. WEIGHTED_Fst; Supplementary Material Table S3. Up_Pi and down_PI; Supplementary Material Table S4. Up_Pi and down_PI; Supplementary Material Table S5. BR_Intersection_gene and BWR_Intersection_gene.
